# Using eHealth to Support Quality of Life and Well-Being in Patients With Lung Cancer: Systematic Review

**DOI:** 10.2196/70510

**Published:** 2025-10-27

**Authors:** Virginia Harrison, Katie Jones, Caroline AC Hyde

**Affiliations:** 1 School of Psychology and Counselling Faculty of Arts and Social Sciences The Open University Milton Keynes United Kingdom; 2 School of Life, Health and Chemical Sciences Faculty of Science, Technology, Engineering and Maths The Open University Milton Keynes United Kingdom

**Keywords:** lung cancer, eHealth, well-being, quality of life, systematic review, mobile phone

## Abstract

**Background:**

Lung cancer (LC) is the leading cause of cancer-related deaths worldwide and has a substantial impact on patients’ quality of life (QoL) and psychological well-being, due to complex physical, emotional, and social challenges. Addressing these needs is critical; yet, many patients go unsupported. eHealth (using information and communication technology to deliver health-related services) offers a scalable way to provide timely, personalized care for people living with LC.

**Objective:**

This review aimed to evaluate the impact of eHealth interventions on QoL and psychological well-being in patients with LC, and characterize the different strategies used.

**Methods:**

A systematic review was conducted following PRISMA (Preferred Reporting Items for Systematic Reviews and Meta-Analyses) guidelines. Literature searches across 6 databases (PubMed, PsycINFO, MEDLINE, Scopus, Web of Science, and CINAHL) were performed between December 2023 and February 2024. Eligible studies included experimental and quantitative designs involving adults (≥18 years) diagnosed with LC. Interventions were required at least 1 eHealth component, and studies had to report outcomes on QoL or psychological well-being. Data extraction focused on study characteristics, intervention details, outcomes, engagement and acceptability metrics. Study quality was assessed using a modified Downs and Black checklist, and a synthesis without meta-analysis was conducted due to study heterogeneity.

**Results:**

A total of 7065 records were screened, with 33 studies meeting inclusion criteria; of these, 30 were suitable for quantitative synthesis, comprising 2654 individual participants and 231 patient-caregiver dyads. eHealth strategies included: patient education (n=2), digital symptom monitoring (n=6), physical activity programs (n=8), psychological support (n=5), nurse-led interventions (n=5), and multicomponent portals or platforms (n=7). For QoL, the most consistent benefits were observed in multicomponent (5/5) and nurse-led (3/3) interventions, followed by physical activity (4/6) and symptom monitoring (4/6) approaches. For psychological well-being, multicomponent (4/4), nurse-led (2/2), and physical activity (6/6) interventions all demonstrated consistent positive effects. Psychological interventions showed mixed effects overall, although mindfulness-based programs (2/2) consistently reduced psychological symptoms. Key factors linked to positive outcomes included personalization, delivery via apps or web-based platforms, longer intervention duration, and clinician involvement. User acceptability was generally high, and engagement was variable, although both were rarely measured.

**Conclusions:**

eHealth interventions can have a positive effect on QoL and psychological well-being for people with LC. Multifaceted programs addressing diverse patient needs were found to be particularly effective. However, variation in study quality, small sample sizes, and inconsistent measurement of engagement and acceptability limit the strength of conclusions. Thus, while eHealth solutions have the potential to address significant gaps in LC care and improve patient outcomes, further research is needed. To realize their full potential, future research should prioritize developing and evaluating tailored, scalable eHealth solutions with robust designs, standardized outcomes, and strategies to enhance patient engagement and implementation in routine care.

**Trial Registration:**

PROSPERO CRD42024509607; https://tinyurl.com/ycst2r8k

## Introduction

Lung cancer (LC) is the leading cause of cancer-related death worldwide [1], with approximately 2.5 million new cases diagnosed each year [2]. Although most patients are diagnosed at an advanced stage (stage III or IV), where 5-year survival rates remain poor [3], recent treatment advances have led to meaningful improvements in survival [4]. As such, a growing number of individuals are living with LC for longer periods, which has important implications for ongoing supportive care into survivorship. A diagnosis of LC may have a substantial impact on a patient’s psychological well-being, as well as their overall quality of life (QoL). While these are related concepts, in the context of this report (and in line with the literature that distinguishes between them [5]), we take QoL to represent the cognitive appraisal a patient makes about their situation in life, while psychological well-being refers more to a patient’s emotional experience or mental health symptoms. However, it is important to consider these 2 concepts in parallel when considering how best to support patient well-being, as psychological symptoms and QoL are closely interlinked in a bidirectional manner in patients with LC [6].

In addition to the distress caused by the diagnosis itself, there are several cancer and treatment-related factors that might negatively impact well-being and QoL in patients with LC. For example, the physical symptoms of LC (eg, breathlessness, pain, and fatigue) and associated paraneoplastic syndromes [7] may functionally impact patients and directly adversely affect their QoL and mental health [8]. Furthermore, both tumors and cancer treatments (including chemotherapeutic agents, immunotherapy, targeted treatments, surgical procedures, and radiotherapy) may cause inflammatory responses and interfere with neuronal function or neurotransmission, which are both biological mechanisms recognized to play a role in the development of psychological disorders [9-11]. Indeed, patients with LC have been found to have significantly higher levels of clinical anxiety and depression [12] and psychological distress [13,14] than both the general population and patients with other types of cancer.

Thus, there is a clear need to support the well-being and QoL needs of this patient group. However, research into well-being–related interventions for patients with LC is lacking. This is problematic, as poor psychological health and well-being may negatively impact patient outcomes. For example, research has found that persistent psychological symptoms are associated with low QoL and poor adherence to anticancer treatments [15,16], which may contribute to high symptom burden [17] and increased mortality [18]. Furthermore, 2 meta-analyses have found a predictive relationship between depression (and, to a lesser extent, anxiety) and mortality in cancer patients [19,20]. Additionally, Andersen et al [21] examined the trajectories of psychological symptoms over 2 years from diagnosis and found that remission of depression (and in 1 model, anxiety) was associated with improved survival, illustrating the potential importance of treating psychological symptoms and supporting well-being in these patients. Similarly, QoL at diagnosis [22] and changes in QoL scores over time [23] have been shown to predict response to treatment, symptom burden, and patient survival.

Despite the apparent importance of well-being and QoL in terms of patient outcomes, this area remains underresearched and undersupported. Across various cancer populations, 2 systematic reviews have found that patients frequently report having unmet psychological needs and a wish for support [24,25]. In LC populations, few receive adequate and readily accessible mental health support, and many are unaware of what well-being–related support is available [26]. As such, it is imperative that we identify better strategies to support them.

eHealth offers a potentially scalable solution that can increase timely access to health-related information, psychoeducation, and support, while also supporting treatment. The World Health Organization [27] defines eHealth as “the use of information and communications technology in support of health and health-related fields.” As such, the modality and content of these types of interventions are extremely heterogeneous. They can include telehealth or telecommunication approaches, use of electronic health records, delivery of online health information and communication (including social media or forum peer-based support), the use of patient-reported outcomes (PROs) to monitor symptoms, health-related video games, and virtual reality platforms. Increasingly, this also includes app or web-based therapies (such as internet-based cognitive behavioral therapy [CBT] and mindfulness) or behavioral change programs, and electronic data capture of objective behavioral or physiological measures via wearable devices (eg, activity trackers such as FitBit [Google LLC], Apple Watch, etc). Across all platforms, eHealth interventions can be guided or unguided and used remotely or in person.

Over the last decade, a significant number of eHealth programs have been developed for various mental health conditions [28] and in an array of different physical health contexts [29,30]. In the general population, eHealth programs designed to support well-being and psychological health have good acceptance [31] and have demonstrated positive outcomes for common mental health disorders [32]. Furthermore, systematic reviews have also produced evidence to suggest that the use of eHealth programs is beneficial in other cancer populations [33]. While several well-being–related eHealth strategies and interventions have been developed to directly help patients with LC [34-36], these have not always been evaluated. One recent small-scale systematic review with patients with LC did show that eHealth is likely to be both acceptable and potentially efficacious with this patient group [37]; however, this review only focused on interventions designed to improve physical functioning and did not include psychological health outcomes alongside QoL. Thus, it is still unclear what types of eHealth interventions are most beneficial to patients with LC and to what extent they can impact QoL and emotional well-being.

The present study aimed to address this gap in knowledge and understanding by evaluating the potential impact of eHealth interventions on QoL and psychological well-being of LC populations. Furthermore, given the heterogeneous nature of eHealth approaches, this systematic review sought to extend previous work by exploring and describing all reported avenues and strategies for eHealth support. Thus, the secondary aim of the review was to characterize the nature of the eHealth interventions and strategies identified and explore their acceptability.

## Methods

### Search Strategy

This systematic review was registered with PROSPERO (International Prospective Register of Systematic Reviews; CRD42024509607). A systematic review of eHealth interventions and programs to support the psychological well-being and QoL of patients with LC was conducted between December 2023 and February 2024 following the PRISMA (Preferred Reporting Items for Systematic Reviews and Meta-Analyses) guidelines [38]. The generation and selection of search terms was informed by preliminary scoping searches to identify commonly used terminology in the LC, eHealth, QoL, and psychological literature, and variant spellings and acronyms were taken into account. A librarian with expertise in systematic review methodology was consulted, and the draft strategies were reviewed by all authors and subject specialists. The literature search was performed using 6 databases: PubMed, PsycINFO, MEDLINE, Scopus, Web of Science, and CINAHL, using combinations of search terms designed to capture a broad range of eHealth resources, including digital and telemedicine or therapy (Table 1 and Multimedia Appendix 1). As we wanted to capture all eHealth studies that measured QoL or psychological well-being outcomes, regardless of whether the impact on well-being was a primary aim, “all text” searches were performed in all cases. Reference lists of identified studies were also examined, and a cited-by search was conducted using Google Scholar to identify further high-quality studies. No restrictions were placed on the publication date.

**Table 1:** Search strategy.

Search	Terms and Boolean operators
1	eHealth OR e-health OR mHealth OR m-health OR digital health OR web-based therapy OR telemedicine OR telehealth OR telepsychiatry OR teletherapy OR health informatics OR electronic OR iCBT OR ccbt OR e-therapy OR e-psychotherapy OR e-counsel* OR cyber-counsel* OR web-counsel* OR e-mental health
2	Internet OR online OR electronic OR cyber OR web OR mobile OR tele OR app OR application
3	Counselling OR counseling OR therapy OR psychiatry OR psychotherapy OR support OR health
4	Wellbeing OR well-being OR quality of life OR anxi* OR depress* OR psychological distress OR mood
5	Lung neoplasm* OR lung carcinoma OR lung cancer OR lung tumour OR lung tumor
Combined search strategy = (1 OR (2 AND 3)) AND 4 AND 5	

### Inclusion or Exclusion Criteria

We included papers based on the Population, Intervention, Comparator, Outcomes, and Settings criteria outlined in Textbox 1. Studies that included participants with various cancers were eligible for inclusion, provided they broke down outcomes by cancer type and the impact on patients with LC could be determined.

Nonexperimental studies (eg, letters, reviews, case reports, guidelines, or protocols), those published outside of scientific journals, and those not published in the English language were excluded. Studies that only focus on the feasibility or technical properties of eHealth tools, or health care management using e-records or health care analytics with no reference to QoL or well-being outcomes were also excluded.

**Inclusion criteria.**

**Inclusion criteria**

- Population: adults (aged older than 18 years) diagnosed with lung cancer.
- Intervention: interventions of any type (including informational, behavioral, therapeutic, self-guided, and clinician-guided interventions, using either individual or group approaches) aimed at patients with lung cancer who have at least 1 essential eHealth component. eHealth was defined as the use of digital systems or information and communication technologies to support health and well-being–related issues.
- Comparator: treatment as usual and waitlist groups (both active and inactive).
- Outcomes: validated scales measuring psychological well-being (eg, depression, anxiety, and psychological distress) and quality of life, including (but not limited to): Quality of Life: EQ-5D, World Health Organization Quality of Life Scale, Functional Assessment of Cancer Therapy, European Organisation for Research and Treatment of Cancer Quality of Life Questionnaire; Anxiety: Depression, Anxiety and Stress Scales, Beck Anxiety Inventory, Hospital Anxiety and Depression Scales; Depression: Depression, Anxiety and Stress Scales, Beck Depression Inventory, Hospital Anxiety and Depression Scales; Psychological distress: Kessler Psychological Distress Scale; Clinical Outcomes in Routine Evaluation; distress thermometer.
- Settings: experimental and quantitative intervention studies (including randomized controlled trials, quasi–randomized controlled trials, and single-arm pre- or poststudies) in any setting.

### Selection Process

Following the removal of duplicates using review management software Rayyan.ai (Rayyan Systems, Inc), all 3 authors conducted the initial screening of titles and abstracts based on the inclusion and exclusion criteria. Given the large number of studies initially identified, each author independently screened one-third of the studies, and a random subset of 10% was reviewed by at least 1 other author. Where there was uncertainty around eligibility, the full text was reviewed by all 3 authors and discussed.

**Figure 1:**
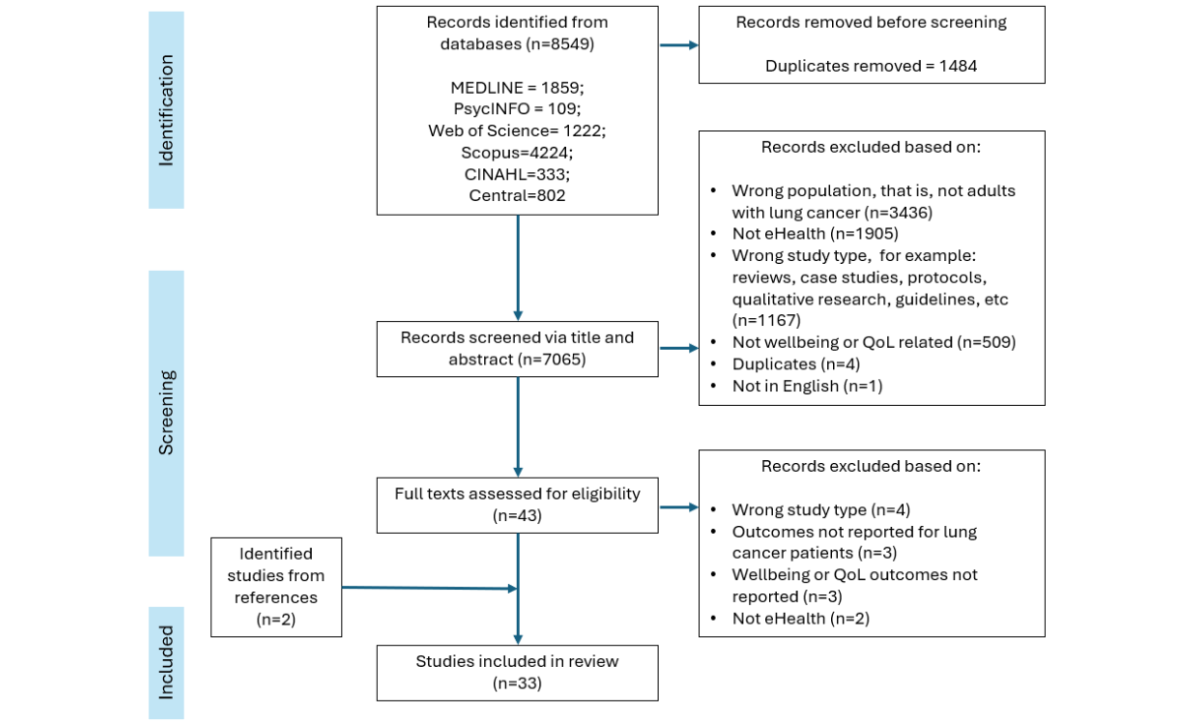
PRISMA systematic review flow diagram. PRISMA: Preferred Reporting Items for Systematic Reviews and Meta-Analyses; QoL: quality of life.

The full texts of potentially eligible studies were then retrieved and assessed by 2 independent reviewers (KJ and VH). Papers were rejected if they did not meet all inclusion criteria or if any exclusion criteria were identified. Any uncertainties or disagreements that arose were resolved through discussion. A PRISMA flow diagram illustrates the study selection process, including the number of studies identified, screened, eligible, and included in the final review (Figure 1).

### Data Extraction

Data from the included studies were extracted independently by 2 reviewers (VH and KJ) using a bespoke standardized data extraction form prepared in Excel (Microsoft Corp). The extracted data included: study characteristics (authors, year of publication, country, study design, study aim, sample size, setting, and data collection points); participant characteristics (age, gender, ethnicity, and cancer type and stage); eHealth program details (nature of the program, mode of delivery [eg, mobile app, web-based platform, telemedicine], LC specificity, degree of clinician input, number of sessions, and duration); outcomes (measures used); acceptability and engagement (patient satisfaction, fidelity, and attrition rates) and key findings. Data extraction was shared evenly, with each reviewer extracting data from 50% of the included studies. To ensure reliability, a random sample (10%) from each reviewer was extracted in duplicate. No conflicts occurred.

### Quality Assessment

The methodological quality of the included studies was independently assessed by 2 reviewers (VH and KJ) using a modified version of the Downs and Black checklist [39]. This method was chosen as it can be used to evaluate both randomized and nonrandomized studies of health care interventions, and it has been ranked in the top 6 quality assessment tools suitable for use in systematic reviews [40]. It has good criterion validity (r=0.90) and good interrater reliability (r=0.75). The checklist comprises 27 items that address several methodological issues, including reporting, external validity, internal validity (bias and confounding), and power. In line with previous studies, we used a modified version of the scale that simplifies the scoring associated with statistical power. While this question carried 5 points in the original scale, more recent studies have awarded this a single point to signify whether a study had sufficient power to detect a clinically significant effect (1) or not (0) [41,42]. Thus, 26 items on the scale are rated as either yes (1), no (0), or unable to determine (0), and 1 (“Are the distributions of principal confounders in each group of subjects to be compared clearly described?”) is rated on a 3-point scale (yes=2, partially=1, and no=0). Item scores are summed to produce a total score ranging from 0-28, with higher values representing better study quality. Using these scores, studies were rated as having poor (≤14), fair (15-19), good (20-25), or excellent (≥26) methodological quality.

### Data Evaluation or Synthesis

A narrative synthesis of the findings was conducted in line with the “synthesis without meta-analysis” guidelines [43]. Due to the heterogeneity in study designs, eHealth programs, and outcome measures, a meta-analysis was not performed. Instead, following recommendations in the Cochrane Handbook for Systematic Reviews of Interventions, vote counting based on direction of effect was used for the quantitative synthesis of results, as synthesizing *P* values was not possible with the available data [44,45]. For the purpose of quantitative synthesis, outcome domains of interest were defined as: QoL (including total QoL scale scores, global QoL measures, and functioning-related QoL subscales), anxiety, depression, and distress. Note that specific physical symptom burden items or subscales (eg, pain, fatigue, or nausea) were not included as independent QoL measures in the effect direction synthesis, as these are often more influenced by disease progression or treatment toxicity than by behavioral or psychosocial interventions. For each outcome domain, we compared the number of studies showing a beneficial effect of the intervention (for pre-post study designs) or a relatively beneficial effect of intervention group (IG) membership (for randomized controlled trials [RCTs] or other studies looking at between group differences in scores over time), with those showing a negative effect. In line with the guidance, we did not take statistical significance or magnitude of effect into account within this quantitative synthesis [44,45]. If a study assessed the same outcome (eg, QoL) using multiple measures (eg, across several subscales), we determined the overall direction of effect based on consistency across those measures. Specifically, when 66% or more showed the same direction of effect (eg, all indicating benefit or all indicating harm), we classified this as the overall effect direction. If fewer than 66% of the measures yielded outcome effects in the same direction, we labeled the effect as mixed. Across all studies, a sign test was used to determine whether the observed pattern of results (ie, the proportion of positive versus negative outcomes) was significantly different from chance.

Results were also grouped thematically according to the type of eHealth intervention under investigation. Unfortunately, statistically investigating heterogeneity through subgroup analyses based on intervention type was not possible due to a lack of sufficient data. Likewise, it was deemed inappropriate to perform separate sign tests for each intervention type due to the lack of power that the relatively small number of studies in each group affords. As such, the impact of the different types of eHealth interventions on patient outcomes, adherence, and QoL was explored more narratively. Greater emphasis was placed on studies that were rated as having higher quality according to the criteria above, and concerns regarding the quality of evidence were reported where necessary.

## Results

### Included Studies

After excluding duplicates, 7065 records were screened against the inclusion and exclusion criteria. This resulted in 7022 exclusions. A total of 43 full-text reports were reviewed in greater depth for additional information, of which 31 were deemed eligible for inclusion. Two additional studies were identified as being eligible from the reference lists of the included reports, resulting in 33 studies in the final review (Figure 1).

### Study Characteristics

Table 2 provides an overview of the studies included in this review, including information about the design of each study and the nature of the eHealth programs under investigation. Recruitment methods were not always reported, but where described, typically occurred in clinical settings as part of routine diagnostic or treatment appointments. Countries that contributed multiple studies to the review were the United States (n=12, 36%), China (n=9, 27%), South Korea (n=3, 9%), and the Netherlands (n=2, 6%). Sample sizes ranged from 16 to 515 participants. The included literature was published between 2014 and 2024, with the majority (n=19, 58%) being published in 2020 or later.

**Table 2:** Study characteristics.

Authors (year) country			Study design		Study aim		Sample size		Relevant outcome measures		Data collection points		Quality score
**Patient education or health literacy interventions**													
	Sun et al (2017) United States [46]	Single-site pilot, quasi-experimental feasibility study with sequential enrollment comparing the IG^a^ with a TAU^b^ CG^c^		To evaluate the feasibility and acceptability of a multimedia self-management intervention to prepare patients and family caregivers for lung surgery.		Patients n=38 (IG=19; CG=19)		Patient QoL^d^: FACT-G^e^; family caregivers QoL: COH-QOL-FCG^f^		Presurgery; discharge from surgery; 2-4 weeks postdischarge		15	
	Benson et al (2022) United States [47]	Single-site pilot, nonrandomized control trial comparing the IG with a TAU CG		To evaluate a preoperative multimedia education tool to improve patient health knowledge, QoL, and satisfaction with care in thoracic surgery.		n=48 (IG=22; CG=26)		QoL: FACT-G, FACT-L^g^ scores		Presurgery; postsurgery		13	
**Symptom monitoring interventions**													
	Billingy et al (2023) Netherlands [48]	Multisite RCT^h^ using a stepped wedge design to compare 2 IGs with a TAU CG		To examine the effect of weekly PRO^i^ symptom monitoring via a reactive approach or active approach on QoL at 15 weeks posttreatment initiation.		n=515 (active IG=160; reactive IG=89; CG=266)		QoL: EORTC QLQ-C30^j^		Baseline: 15 weeks		24	
	Dai et al (2022), China [49]	Multisite RCT comparing the IG with a TAU CG		To identify the benefits of using an electronic PRO-based intervention with an alert system after LC^k^ surgery on QoL		n=134 (IG=65; CG=69)		QoL: SIQOL^l^ (single item); Ψ^m^: MDASI-LC^n^		Baseline, daily during hospital stay, twice weekly postdischarge for 4 weeks		23	
	Prasongsook et al (2022) Thailand [50]	A single-site pilot validity RCT comparing the IG with a TAU CG		To develop and evaluate the validity of an online PRO-based mobile app during the COVID-19 pandemic era.		n=33 (IG=17; CG=16)		QoL: FACT-L		Baseline; 3-4 weeks; 3 months		23	
	Yount et al (2014) United States [51]	A multisite, nonblinded RCT comparing patients completing online monitoring with (IG) and without (CG) automatic reporting		To evaluate whether technology-based weekly symptom monitoring with automated reporting of problematic symptoms to HCPs^o^ reduces on-treatment symptom burden in comparison to monitoring alone.		n=253 (IG=123; CG=130)		QoL: FACT-G; Ψ: SDS^p^		Baseline; 3; 6; 9; 12 weeks		22	
	Denis et al (2017) France [52]	Multisite phase III RCT, comparing the IG with TAU CG		To compare the outcomes of patients with web-mediated follow-up based on weekly PROs (IG) with standard follow-up (CG).		n=133 (IG=67; CG=66)		QoL: FACT^q^		Baseline; 6 months (but weekly PROs collected)		20	
	Maguire et al (2015) United Kingdom [53]	A multisite pre- or post, single-arm, mixed methods feasibility study		To explore the feasibility and acceptability of an app-based symptom management program with patients receiving radiotherapy; and to assess changes in the outcomes of patients using the app.		n=16		QoL: FACT-L; Ψ: STAI^r^, ESAS^s^ (anxiety and depression subscales)		Baseline; end of treatment (exact time not reported)		10	
**Physical activity or pulmonary rehabilitation interventions**													
	Ha et al (2023) United States [54]	A phase-IIb, parallel-group, pilot RCT comparing the IG with an active CG		To test the feasibility, acceptability, safety, and potential efficacy of a telemedicine-based physical activity or pulmonary rehabilitation intervention on a range of PROs.		n=28 (IG=14; CG=14)		QoL: SGRQ^t^; Ψ: GAD-7^u^		Baseline; 6 weeks; 12 weeks		20	
	Bade et al (2021) United States [55]	Single-site pilot RCT comparing the IG with a TAU CG		To examine the effects of the intervention on physical activity; dyspnea, QoL, depression, and biomarkers.		n=40 (IG n=20; CG n=20)		QoL: EORTC-QLQ-C30; Ψ: PHQ-9^v^		Baseline; 12 weeks		19	
	Ji et al (2019) South Korea [56]	Single-site RCT comparing two IGs: (1) a fixed exercise group, (2) a fixed-interactive exercise group (no CG)		To investigate the effects of a personalized pulmonary rehabilitation program on patients with NSCLC^w^.		n=64; (IG=32; CG=32)		QoL: EQ-VAS, EQ-5D		Baseline; 6 weeks; 12 weeks		19	
	Granger et al (2018) Australia [57]	Single-site pre- or postfeasibility study		To explore the effectiveness of a physical activity self-management program aiming to increase activity levels of patients undergoing surgery for LC.		n=37		QoL: EORTC QLQ-C30; Ψ: HADS^x^		Baseline; 8 weeks		17	
	Coats et al (2019) Canada [58]	Single-site pilot pre- or post single-arm feasibility study		To investigate the feasibility, adherence, satisfaction, and preliminary efficacy of a home-based telerehabilitation program in patients with unresectable thoracic neoplasia receiving chemotherapy.		n=5		QoL: EORTC QLQ-C30; Ψ: HADS		Baseline; 8 weeks		16	
	Lei et al (2022) China [59]	Single-site parallel, pseudorandomized, controlled trial comparing the IG with a TAU CG		To determine the effect of the exercise prescription on QoL, depression, and anxiety in patients with LC.		n=52		QoL: EORTC-QLQ-C30; Ψ: ZSDS^y^, Zung Self-Rating Anxiety Scale		Baseline; 8 weeks		15	
	Chen et al (2024) China [60]	Single-site retrospective case-control study comparing the IG with a TAU CG		To investigate the effect of an exercise-based pulmonary rehabilitation program on pulmonary function and QoL of patients with LC.		n=100 (IG=51; CG=49)		QoL: EORTC-QLQ-C30; Ψ: ZSDS, Zung Self-Rating Anxiety Scale		Pre and postintervention (exact times not reported)		12	
	Lafaro et al (2020) United States [61]	Pilot pre- or postintervention		To determine the feasibility and acceptability of a personalized telehealth perioperative physical activity intervention for older patients of lung and gastrointestinal cancer surgery and their caregivers, and describe the trajectories of recovery and PROs before and after surgery and intervention.		n=34, of which 18 had LC		Ψ: distress thermometer		Baseline (7-14 days before surgery); within 24 hours of discharge; 2-4 weeks post discharge		12	
**Psychological well-being interventions**													
	Mosher et al (2016) United States [62]	Multisite pilot RCT comparing the IG to an active CG (receiving education and support)		To examine the preliminary efficacy of telephone-based support for symptomatic patients with LC and their family caregivers.		n=106 dyads (IG=51; CG=55)		Ψ: PHQ^z^; GAD-7		Baseline; 2 and 6 weeks		22	
	Tian et al (2023) China [63]	Single-site, single-blinded, longitudinal, RCT comparing the IG to TAU CG		To evaluate the efficacy of an MBSR^aa^ program on psychological distress in patients with LC and elucidate its mechanisms.		n=175 (IG=83; CG=92)		Ψ: distress thermometer		Baseline; 1 and 3 months		22	
	Mosher et al (2019) United States [64]	Multisite pilot RCT comparing the IG to an active CG (receiving education and support)		To examine the feasibility and preliminary efficacy of telephone-based ACT^ab^ for symptomatic, advanced patients with LC and their distressed family caregivers.		n=50 dyads (IG=25; CG=25)		Ψ: PROMIS^ac^, distress thermometer		Baseline; 2 and 6 weeks		21	
	Huang et al (2021) China [65]	Multisite RCT comparing patients assigned to individual (ICMT^ad^) or group (GCMT^ae^) therapy with a TAU CG		To explore the effects of individual and group magnanimous therapy on the emotional, psychosomatic, and immune functions of patients with advanced LC.		n=116 (ICMT=40; GCMT=36; CG=40)		Ψ: PSSCP^af^, HADS		Baseline; 2 weeks posttherapy		19	
	Milbury et al (2020) United States [66]	Single-site pilot RCT, comparing the IG with an active control (using supportive expression: SE^ag^), a TAU CG		To examine the feasibility of a CBM^ah^ intervention via videoconference delivery.		n=75 patient-caregiver dyads (CBM=26; SE=24, CG=25)		Ψ: distress thermometer, CES-D^ai^, IES^aj^		Baseline; 4 and 12 weeks		19	
**Nurse-led interventions**													
	Reinke et al (2022) United States [67]	Multisite RCT comparing the IG with a TAU CG		To test the effect of a nurse-led, telephone-based palliative care intervention among patients with newly diagnosed any-stage LC.		n=151		QoL: FACT-L		Baseline; 12 weeks		20	
	Hintistan et al (2017) Turkey [68]	Single-site quasi-experimental study comparing the IG with a TAU CG		To determine the therapeutic effects of nurse telephone follow-up for patients with LC on performance status, symptomatology, and QoL.		n=60 (IG=30; CG=30)		QoL: FLIC^ak^ Ψ: ESAS (including anxiety and distress)		First chemotherapy cycle, midcycle, last cycle		15	
	Sherry et al (2017) United States [69]	Single-site pilot pre- or postintervention study		To pilot distress screening using the distress thermometer and evaluate the effect of a patient education pamphlet and coaching call on distress.		n=41		Ψ: distress thermometer		Baseline; 1-3 weeks		14	
	Shi and Yang (2023) China [70]	Single-site RCT comparing the IG with a TAU CG		To investigate the effectiveness of a synthetic nursing intervention on the QoL and self-care ability of athletic patients retired due to LC undergoing surgery.		n=72		QoL: FACT-L		Pre and postintervention (exact times not reported)		14	
	Li et al (2016) China [71]	Single-site noninferiority, retrospective case-control analysis, comparing the IG with a TAU CG		To explore the efficacy of internet-based intervention on the QoL of patients with chronic postsurgical pain.		n=81 (IG=41; CG=40)		QoL: SF-36^al^		Baseline; 1 and 3 months posttreatment		10	
**Multicomponent programs and patient portals**													
	Huang et al (2019) Taiwan [72]	Single-site RCT comparing the IG with a TAU CG		To evaluate the effects of a web-based health education program on QoL and symptom distress in patients with advanced NSCLC awaiting chemotherapy.		n=55 (IG=27; CG=28)		QoL: EORTC QLQ-C30; Ψ: SDS		Baseline; 1; 2; and 3 months after beginning chemotherapy		23	
	Sui et al (2020) China [73]	Single-site RCT comparing IG with a CG receiving only a simple session of education and rehabilitation guidance before hospital discharge		To explore whether a WeChat-based education and rehabilitation program affected anxiety, depression, QoL, loss of follow-up, and survival profiles in patients with NSCLC after undergoing surgical resection.		n=200 (IG=100; CG=100)		QoL: EORTC-QLQ-C30; Ψ: HADS		Baseline; 3; 6; 9; and 12 months		20	
	Park et al (2019) South Korea [74]	Single-site pilot single-arm pre- or postintervention study		To determine the feasibility and efficacy of smartphone app-based pulmonary rehabilitation on exercise capacity, symptom management, and QoL in patients with advanced LC undergoing chemotherapy.		n=90		QoL: EORTC QLQ-C30, Ψ: GAD-7, PHQ-9		Baseline, then every 4-6 weeks at routine clinic visits, and 12 weeks		19	
	Jiang et al (2024) China [75]	Single-site RCT comparing the IG with a TAU CG		To evaluate the effects of a mobile health-based management for patients with NSCLC on pulmonary function and QoL		n=60 (IG=30; CG=30)		QoL: EORTC QLQ-30		Baseline; 1 week, 2 weeks; then, 1, 6, and 12 months		18	
	Yang et al (2022) South Korea [76]	Single-site, single-arm pre- or postfeasibility or pilot study		To evaluate the efficacy of a remote health care program for patients with LC and determine whether the program helped improve QoL, cardiorespiratory endurance, and muscle strength.		n=50		QoL: EORTC QLQ-C30		Baseline; 6 and 12 weeks		17	
	Groen et al (2017) Netherlands [77]	Single-site, pre- or postfeasibility study		To evaluate the feasibility and usability of a patient portal and generate preliminary evidence on its impact on patient activation, QoL, and physical activity.		n=37		QoL: SF-36		Baseline; 4 months		13	
	Kneuertz et al (2020) United States [78]	Pilot pre- or postevaluation study		To evaluate the use of a mobile app for patient engagement and PROs assessment following robotic LC surgery.		n=50		Nonvalidated daily measures of anxiety and mood		Anxiety reported daily (during app use) for 30 days		11	

^a^IG: intervention group.

^b^TAU: treatment as usual.

^c^CG: control group.

^d^QoL: quality of life.

^e^FACT-G: Functional Assessment of Cancer Therapy—General.

^f^COH-QOL-FCG: City of Hope Quality of Life Tool—Family Caregivers.

^g^FACT-L: Functional Assessment of Cancer Therapy—Lung.

^h^RCT: randomized controlled trial.

^i^PRO: patient-reported outcomes.

^j^EORTC QLQ-C30: European Organisation for the Research and Treatment of Cancer Quality of Life Questionnaire.

^k^LC: lung cancer.

^l^SIQOL: Single Item Quality of Life Scale.

^m^Ψ: psychological.

^n^MDASI-LC: MD Anderson Symptom Inventory—Lung Cancer.

^o^HCP: health care professional.

^p^SDS: Symptom Distress Scale.

^q^FACT: Functional Assessment of Cancer Therapy.

^r^STAI: State-Trait Anxiety Inventory.

^s^ESAS: Edmonton Symptom Assessment System.

^t^SGRQ: St. George’s Respiratory Questionnaire.

^u^GAD-7: Generalized Anxiety Disorder Questionnaire.

^v^PHQ-9: Patient Health Questionnaire.

^w^NSCLC: non-small cell lung cancer.

^x^HADS: Hospital Anxiety and Depression Scales.

^y^ZSDS: Zung Self-Rating Depression Scale.

^z^PHQ: Patient Health Questionnaire.

^aa^MBSR: mindfulness-based stress reduction.

^ab^ACT: Acceptance and Commitment Therapy.

^ac^PROMIS: Patient-Reported Outcomes Measurement Information System.

^ad^ICMT: individual computer-based magnanimous therapy.

^ae^GCMT: group computer-based magnanimous therapy.

^af^PSSCP: Psychosomatic Status Scale for Cancer Patients.

^ag^SE: supportive expression.

^ah^CBM: couple-based meditation.

^ai^CES-D: Center for Epidemiological Studies Depression.

^aj^IES: Impact of Event Scale.

^ak^FLIC: Functional Living Index-Cancer.

^al^SF-36: Short Form Health Survey.

All but 1 study [61] included solely patients with LC, and they ranged in terms of the stage of disease that they included: 5 (15%) included only early stage [49,54,68,73,77], 8 (24%) included only advanced disease [51,52,58,64-66,69,72], 12 (36%) included all stages [46,48,55-57,59,60,62,63,67,74,76], and 8 (24%) studies failed to report disease stage [47,50,53,61,70,71,75,78]. Studies also varied in terms of their methodology: 17 (52%) were described as pilot or feasibility studies or were single-arm pre-post studies; 13 (39%) were full-scale RCTs; 2 (6%) used retrospective case-control methods; and 1 (3%) used a nonrandomized, quasi-experimental design (Table 2). Additionally, 28 (85%) studies focused solely on patient participants, while 5 (15%) studies also included caregivers or loved ones (although only patient-related outcomes are included in this review).

### Quality Appraisal

A quality rating was assigned to all 33 studies and is provided in Table 2. Overall, in terms of methodological quality, none of the studies were rated as excellent, 12 (36%) studies were rated as good [48-52,54,62-64,67,72,73], 12 (36%) studies as fair [46,55-59,65,66,68,74-76], and 9 (27%) studies as poor [47,53,60,61,69-71,77,78].

### PROs

Most studies (n=26; 79%) focused on QoL, specific psychological symptoms (including depressive and anxiety symptoms), or psychological distress as primary outcomes [46-48,50,54-56,58-60,62-75,77,78]. These measures were secondary outcomes in the remaining 7 (21.21%) studies [49,51-53,57,61,76].

As illustrated in Table 2, QoL was measured in 25 (76%) studies. Seven different validated QoL measures were used: 11 studies used the EORTC QLQ-C30 (European Organisation for the Research and Treatment of Cancer Quality of Life Questionnaire), 8 used the Functional Assessment of Cancer Therapy Scale, 2 used the Short Form Health Survey, 1 used both the EQ-5D and the EQ-VAS, 1 used the Functional Living Index-Cancer, 1 used St. George Respiratory Questionnaire, and 1 used the single item Quality of Life scale.

In terms of specific psychological symptoms, depression was measured in 12 (36%) studies, and anxiety in 13 (39%) studies. The most frequently used measure was the Hospital Anxiety and Depression Scales (n=4) [79]. Four further scales were used to assess depression: 3 used the Patient Health questionnaire [80], 2 used Zung Self-Rating Depression Scale [81], 1 used the Patient-Reported Outcomes Measurement Information System Depression items [82], 1 used the depression item of the Edmonton Symptom Assessment System (ESAS) [83], and 1 used the Center for Epidemiological Studies Depression [84]. A further 4 validated scales measured anxiety: 3 studies used the Generalized Anxiety Disorder Questionnaire [85], 2 used the Zung Self-Rating Anxiety Scale [86], 1 used the Patient-Reported Outcomes Measurement Information System Anxiety items [82], and 1 used the State-Trait Anxiety Inventory [87]. Additionally, 2 studies used the anxiety item from the ESAS [83]; and 1 study used nonvalidated daily measures of anxiety and mood.

Psychological distress was measured in 10 (30%) studies. A total of 5 studies used the distress thermometer [88] (1 in combination with the Impact of Event Scale to assess cancer-related stress) [89], 2 studies used the Symptom Distress Scale [90], 1 used the ESAS well-being subscale [83], and 1 used the Psychosomatic Status Scale for Cancer Patients [91]. An additional study used the MD Anderson Symptom Inventory [92] to infer affective interference.

### Intervention Types

**Figure 2:**
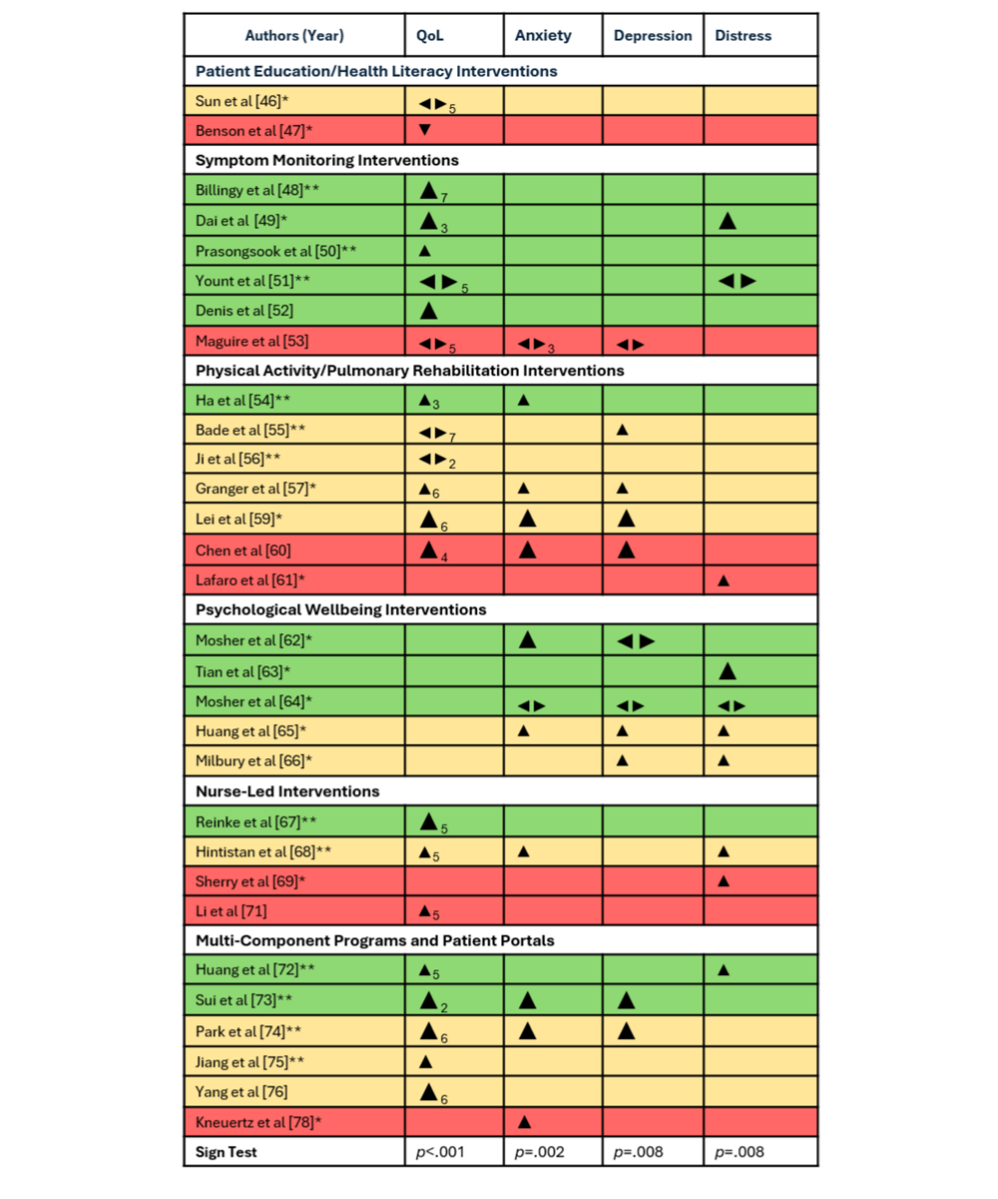
Effect direction plot [46-57,59-69,71-76,78]. Effect direction: upward arrow ▲ = positive health impact, downward arrow ▼ = negative health impact, sideways arrow ◂▸ = no change, mixed effects, or conflicting findings. Sample size: large arrow indicates n of 50‐300 in IG; small arrow indicates n <50 in IG. Subscript numbers: number of outcomes within each category is 1 unless indicated by a subscript number beside the effect direction arrow. Study quality: denoted by row color, such that green = high quality, yellow = fair quality, and red = poor quality. Study duration: * indicates intervention was <12 weeks; ** indicated intervention was ≥ 12 weeks. IG: intervention group; QoL: quality of life.

Reflecting the heterogeneous nature of eHealth programs, the reviewed studies used interventions that varied significantly from 1 another in both scope and technological basis. Of the included studies, 14 aimed to improve health literacy via education about LC symptoms, treatment-related side effects, or symptom management [46,47,54,55,57,67,69,71-75,77,78]; 13 provided psychological or well-being support [62-66,68-74,76]; 13 were used for symptom monitoring [48-53,67,68,72,75-78]; 12 aimed to improve physical ability, often as a form of pulmonary rehabilitation [54-61,73,74,76,77]; and 3 provided access to medical records or laboratory results [74,75,77]. Categories were not mutually exclusive, with many studies using eHealth to deliver multiple avenues of support simultaneously. To better understand how different intervention characteristics related to patient outcomes, results for each study have been grouped according to the interventions’ main functions (Tables 2 and 3 and Figure 2).

**Table 3:** Description of interventions.

Authors			eHealth type		Intervention description		Overall duration
**Patient education or health literacy interventions**							
	Sun et al [46]	Video (shown in hospital setting), handbook, and 2× telephone support sessions with a clinician		Multicomponent intervention focusing on proactive planning, knowledge enhancement, self-efficacy, and activation. Sessions focused on what to expect immediately before, during, and after surgery and included postdischarge telephone calls.		4-7 days before surgery until 4-6 weeks post surgery	
	Benson et al [47]	A multimedia education tool delivered in-person alongside standard care		Preoperative, personalized interactive education about LC^a^ surgery, including scan images and an interactive 3D model with animated video clip simulations to demonstrate the planned procedure, with personalized annotations made in real time by the surgeon.		One education session	
**Symptom monitoring interventions**							
	Billingy et al [48]	PRO^b^-based mobile app and email alerts, followed up in the IG^c^ with triage, advice, and from HCPs^d^ as required		Two IGs: both received a PRO-based symptom-reporting app. In the reactive group, patients received an alert notification and secure email containing the advice to contact the hospital if they scored over a preset threshold. In the active group, HCPs received an alert via a (secure) email instructing them to contact the patient.		15 weeks	
	Dai et al [49]	A data platform (REDCap^e^; Vanderbilt University), an electronic PRO system, and a communication service app		While all patients reported symptoms via an electronic PRO-based system, patients in the IG also had real-time electronic alerts sent to treating surgeons if they reported scores above a preset threshold. Surgeons responded within 24 hours with personalized interventions.		Median in-patient duration=5 days; plus 4 weeks post discharge	
	Prasongsook et al [50]	Mobile app with clinician response via app or phone call		LC-specific app for self-reporting self-assessments of QoL^f^ and side effects from systemic treatment. Clinicians responded to severe PROs with instant advice.		3 months	
	Yount et al [51]	Telephone-based IVR^g^ technology		All patients reported symptoms via an automated telephone system for monitoring PROs. In the IG, symptom reports were also automatically delivered to HCPs for further consideration, who directly responded to patients when problematic symptoms were reported.		12 weeks	
	Denis et al [52]	Electronic PROs reporting and web-mediated follow-up of symptoms		Patients reported symptoms electronically weekly. In the IG, an alert email was automatically sent to the oncologist when PROs exceeded a predefined criterion. Clinicians responded to alerts as needed.		Various	
	Maguire et al [53]	A remote patient monitoring mobile phone app		Patients completed a daily questionnaire on a mobile app, and symptom data were sent in real time to the study server. Following an automated risk analysis of responses, self-care advice directly related to the severity of reported symptoms was sent to patients’ phones. Where symptoms were of clinical concern, alerts were sent out to HCPs’ pagers.		7 days a week for the duration of their radiotherapy treatment and 1 month after treatment	
**Physical activity or pulmonary rehabilitation interventions**							
	Ha et al [54]	Telehealth and wearable technology		Participants in the IG received an intervention comprising essential components of pulmonary rehabilitation (ie, exercise training, education, and behavioral support), delivered via 6 live tele-visits over 12 weeks, with inspiratory muscle training and wearable activity trackers.		30-60 minutes a session over 12 weeks	
	Bade et al [55]	Wearable activity tracker, mobile app, and text messages		Education about the benefits of physical activity in LC; individualized walking goals; twice daily gain-framed text messages. Initial in-person education (15 minutes) provided by the study team.		12 weeks	
	Ji et al [56]	Mobile app, online database, and wearable pulse oximeter		All patient-generated health data, such as 6MWT^h^ results, rehabilitation exercise progress, heart rate, and breathing difficulty levels, are sent from the app to a central database monitored by HCPs.		12 weeks	
	Granger et al [57]	Telehealth and wearable technology		A home aerobic exercise program was taught in an initial face-to-face consultation and followed up with weekly telephone consultations with a physiotherapist, supported by patient education, behavior change techniques, and provision of an activity monitor.		8 weeks	
	Coats et al [58]	Telerehabilitation station including touch screen computer, webcam (for videoconferencing), and biomechanical sensors for physiological measurements		15 sessions of telerehabilitation supervised in real time by a clinical physiologist or cancer exercise trainer and 9 unsupervised sessions. Patients performed interactive gamified exercises set to music, and biometric data was continuously collected and sent to their HCP.		8 weeks	
	Lei et al [59]	Videocall and social media		Exercise intervention. Instructions given in person, brochures, and online videos. Progress checks via video call and social media.		8 weeks	
	Chen et al [60]	Wearable technology pedometer monitored by a clinician		The IG received a pulmonary rehabilitation exercise program based on a wearable device pedometer. Patients were directed by a clinician to perform a range of simple physical and breathing exercises, dependent on their ability.		Not stated	
	Lafaro et al [61]	Telehealth and a wearable pedometer with coaching provided by a trained physical or occupational therapist		One-on-one coaching to optimize physical and psychological functioning before and after surgery, including classic behavioral change strategies (eg, goal setting, identifying challenges or barriers to physical activity, and problem-solving to overcome the challenges or barriers) and skills building.		Minimum 7 days before surgery, up to 2-4 weeks post discharge	
**Psychological well-being interventions**							
	Mosher et al [62]	Telephone		A telephone-based intervention with patients and caregivers jointly attending 4 × 45-minute clinician-led sessions. Includes both evidence-based CBT^i^ and EFT^j^ to address patient and caregiver anxiety and depressive symptoms, and patient pain, fatigue, and breathlessness.		4 weeks	
	Tian et al [63]	WeChat app and telephone		A modified MBSR^k^ program delivered under the guidance and supervision of a psychologist qualified as a mindfulness trainer who provided supervision twice weekly using telephone or WeChat.		4 weeks	
	Mosher et al [64]	Telephone		Dyadic (patient-caregiver) and individual ACT^l^ sessions delivered via one 50-minute telephone session per week for 6 weeks.		6 weeks	
	Huang et al [65]	Computer-based therapy presentation		IG groups received computer-based magnanimous therapy-based presentations, facilitated by a therapist. In the ICMT^m^ group, the therapist used the presentation to treat 1 patient at a time. In the GCMT^n^ group, the therapist focused on inspiring a positive interaction effect in the group.		2 weeks	
	Milbury et al [66]	Facetime (Apple Inc)		Patients and spouses together attended 1 session per week for 4 weeks (60 minutes each; total of 240 minutes), including intrapersonal (ie, mediations) and interpersonal (ie, emotional sharing) exercises.		4 weeks	
**Nurse-led interventions**							
	Reinke et al [67]	Telephone		Symptom assessment and management, person-centered care plans, and education on LC treatments. Nurses made an initial visit to the patient, followed by 8 phone calls over 12 weeks or until their cancer treatment was complete.		12 weeks or until completion of primary treatment	
	Hintistan et al [68]	Telehealth		Patient received a call from a nurse in the first week after each chemotherapy session (a total of 6 calls) to determine current well-being and symptoms, give advice on symptom management, support physical and psychosocial functioning, facilitate communication, and signpost to other support.		One call every ~3-4 weeks, of approximately 10-25 minutes depending on the participant’s needs.	
	Sherry et al [69]	Tablet screening or assessment and telephone coaching		Patients disclosing high levels of distress on the digital distress thermometer were provided an education pamphlet and follow-up coaching calls.		1-3 weeks (from 1 routine clinic visit to the next)	
	Shi and Yang [70]	WeChat-based program		Health education as well as psychological intervention was provided alongside other forms of intervention—a WeChat platform provided information about LC through short videos, animation, and posters.		Not reported	
	Li et al [71]	Telephone and internet		IG received the same therapy as the CG^o^ without going to the hospital. Instead, relationships with patients were managed via telephone and the internet, and psychological support and pain-related education were conducted remotely.		Not reported	
**Multicomponent programs and patient portals**							
	Huang et al [72]	Web-based program, including notification via text messages		Six-part web-based program, comprising symptom monitoring, explanations of laboratory data, education about LC, symptom management including chemotherapy- or radiation-induced symptom distress, supportive care consisting of emotional support, available social resources, and patient stories, and space for patients to ask open questions.		3 months	
	Sui et al [73]	WeChat-based program		WeChat-based education rehabilitation program, which included disease-related health education, rehabilitation exercise guidance, daily activity supervision, and psychological support provided by trained nurses.		Health education: 12 weeks; exercise guidance: 40 weeks; activity supervision: 12 months; psychological support: 12 months	
	Park et al [74]	Wearable device connected with an app		The app provided a structured animation video guiding physical activity. Patients were instructed to use the device and app during their exercise, and their activity was shared with the attending physician in real time. The app also contained patients’ individual laboratory results and CT images, information on LC and treatment side effects, a to-do list, an in-app chat service, and facilitated CNS^p^-led counseling. Push notifications were sent to patients to remind them to exercise, take medication, and perform other daily tasks.		12-week rehabilitation program	
	Jiang et al [75]	WeChat-based program prompting clinician interventions		A patient portal comprising medical records, nurse-patient communication, message reminders, disease and treatment-related education, and symptom or data monitoring. If a patient’s data exceeds a prespecified threshold, an alert is sent to the patient and the responsible physician, who proactively intervenes.		Up to a year	
	Yang et al [76]	Smartphone app; telehealth		The app allowed participants to objectively digitally monitor their blood pressure, heart rate, oxygen saturation, and FEV^q^, as well as provide self-reported information about their symptoms daily. The app also included a prescribed exercise program (with guided videos) and a diet program. Patients also received weekly health counseling via telephone.		Not stated	
	Groen et al [77]	Online portal		An online interactive portal that features personalized patient education material, appointment diaries, access to electronic medical records, PROs, and related feedback (ie, a graphical and tabular overview of scores), and tailored physical activity advice.		4 months, but noncommittal use permitted	
	Kneuertz et al [78]	Mobile app including space for direct provider-patient communication		Cloud-based platform for providers to educate, engage, and monitor patients throughout the pre- and postoperative period. The app includes reminders, task lists, an education library, progress tracking, and surveys for collecting PROs, with content customized to match existing thoracic surgical care pathways and clinical protocols.		Presurgery to 30 days post discharge	

^a^LC: lung cancer.

^b^PRO: patient-reported outcomes.

^c^IG: intervention group.

^d^HCP: health care professional.

^e^REDCap: Research Electronic Data Capture.

^f^QoL: quality of life.

^g^IVR: interactive voice response.

^h^6MWT: 6-minute walk test.

^i^CBT: cognitive behavioral therapy.

^j^EFT: emotion focused therapy.

^k^MBSR: mindfulness-based stress reduction.

^l^ACT: acceptance and commitment therapy.

^m^ICMT: individual computer-based magnanimous therapy.

^n^GCMT: group computer-based magnanimous therapy.

^o^CG: control group.

^p^CNS: clinical nurse specialist.

^q^FEV: forced expiratory volume.

### Quantitative Synthesis

#### Direction of Effect

The directions of effect can be seen in Figure 2, created following guidance from Boon and Thomson [45]. Regardless of whether a lower score indicates an improved outcome (as in psychological measures), or whether a higher score indicates better performance (as in QoL measures), findings are represented so that an upwards arrow indicates a favorable effect of the intervention or IG; a downwards arrow indicates a negative impact of the intervention or better performance in the control group; while horizontal arrows indicate no change or mixed effects.

Three studies that are described in Tables 2 and 3 were excluded from the quantitative synthesis, as 2 failed to report descriptive or effect-related statistics [58,77], while another contained conflicting information between the statistics reported and descriptive interpretation in the text, making it impossible to infer the true effect direction [70]. Of the remaining 22 studies that measured QoL, 16 showed a positive effect, 1 showed a negative effect, while 5 reported conflicting or unclear effects. For anxiety, 10 studies reported a positive effect direction, none reported a negative effect, and 2 reported mixed results. For depression, 8 studies reported a positive effect direction, none were negative, and 3 were mixed. Further, for distress, 8 were positive, none were negative, and 2 were mixed. In all cases, the sign test showed the pattern of results significantly favored the intervention (*P*s range from <.001 to .008). However, from looking at the effect direction plot, it is clear that the study results varied by the nature of the intervention studied. As such, patient outcome findings are broken down narratively by category below.

#### Patient Education

Two studies focused solely on patient education as a means of improving health literacy or self-efficacy around health management [46,47]. Both studies had a rather narrow focus: preparing patients for LC surgery and the after-effects. Despite using different approaches (Sun et al [46] investigated the use of a multicomponent perioperative intervention spread over several weeks, while Benson et al [47] used an intervention that was delivered in a single session), the direction of effects revealed that results were either negative or mixed; and neither found a significant effect on overall QoL. Taken together, these studies suggest that education alone may not be enough to impact overall QoL. However, as both studies had relatively small sample sizes and were rated as poor quality, the conclusions that can be drawn from these studies are limited. Furthermore, the impact on psychological outcomes could not be assessed, as neither study included psychological measures.

#### Symptom Monitoring

Six studies focused on the impact of stand-alone PRO-based symptom monitoring technologies on QoL [48-53]. Five used RCT methodologies [48-52] and were rated as good quality, although 1 was a pilot study [50]. In all studies, patients used symptom management programs to electronically report their symptoms. If symptoms exceeded a predefined threshold, alerts were sent to their oncology teams, who proactively responded to their needs in an individualized manner. Four of the studies used online reporting, and all found a positive impact on either QoL overall or its subscales. In contrast, Yount et al [51] used a telephone voice recognition system for symptom reporting and found no consistent effect on QoL. Additionally, a single-arm pre-post study [53] found no consistent improvement in QoL following online symptom reporting. However, this latter study had a small sample size (n=16) and was rated as poor quality, limiting the conclusions that can be drawn.

Only 3 studies measured the impact of the intervention on psychological outcomes. While 1 good quality study found reduced affective interference (interpreted as reduced psychological distress) [49,93], 2 studies found no consistent evidence of a positive effect on either psychological distress [51], depression, or anxiety [53].

#### Physical Activity Interventions

Seven studies investigated the use of physical activity and pulmonary rehabilitation interventions in patients with LC. Six used a combination of personalized exercises or goal-setting along with wearable technology to monitor activity [54-57,60,61]. A seventh study [59] delivered a nonpersonalized exercise program at home, using prespecified online videos and training instructions. Two interventions were entirely home-based [54,56], and 3 were home-based with an initial face-to-face element, varying from a single 15-minute session [55] or an initial consultation [57] to 2 weeks of supervised exercise [59]. Telemedicine was also used in 4 studies: Lei et al [59] had therapists checking on progress twice a week via video call or other social media; while 2 studies [54,61] delivered the entire intervention via live telehealth session (largely using videoconferencing software). In contrast, 1 program occurred in a hospital setting [60].

Of the studies that measured QoL (n=6), the majority (n=4; of which 1 was good-, 2 were fair-, and 1 was of low-quality) found a significant positive impact [54,57,59,60]. An additional study [56] produced mixed results, only finding a positive effect on QoL as measured by the EQ-VAS, but not the EQ-5D. Additionally, Bade et al [55] found a positive impact on global and role function, but found a negative effect for physical, emotional, cognitive, and social QoL subscales.

Of the 6 studies that measured psychological outcomes, 4 measured depression [55,57,59,60], 4 measured anxiety [54,57,59,60], and 1 measured psychological distress [61]. All were rated as having fair-to-good quality, except 2, which were considered poor [60,61]. All found a reduction in psychological symptoms; 2 of which were statistically significant [59,61].

#### Psychological Interventions

Five studies described interventions that specifically focused on improving psychological well-being. These interventions varied in their theoretical frameworks and modes of delivery. Two studies used mindfulness-based approaches: 1 using a modified Mindfulness-Based Stress Reduction program delivered via WeChat and telephone [63], and the other using a couple-based meditation intervention delivered via videoconferencing [66]. Two further studies conducted by Mosher et al [62,64] used dyadic models to deliver therapist-led sessions via telephone, 1 focused on CBT and Emotion-Focused Therapy, and the other on acceptance and commitment therapy (ACT). A fifth study [65] investigated the use of computerized Magnanimous Therapy, delivered either individually or in groups during a patient’s hospitalization. Across these studies, program durations ranged from 2 to 6 weeks, and all involved a significant degree of clinician input, typically through guided sessions led by psychologists or trained therapists. Three of the studies were rated as being of good quality [62-64], while 2 were considered fair [65,66].

In terms of patient outcomes, none of these papers reported QoL data, and therefore impact on patient QoL cannot be assessed. In contrast, psychological outcomes were reported in all cases, although the outcome measures used differed across the studies, and findings were mixed. For example, of the 4 studies that measured depression [62,64-66], 2 found a reduction in symptoms [65,66], while no consistent effect was found in the others [62,64]. Of the 3 studies that measured anxiety [62,64,65], 2 found a positive effect of the intervention [62,65], while 1 found no effect [64]. Of the 4 studies that measured psychological distress [63-66], 3 reported improvements [63,65,66], while 1 found conflicting results [64]. However, it is of note that the 2 studies that found inconsistent effects across psychological outcome measures [62,64] may have been affected by power issues and the use of an active (and possibly therapeutic) control condition.

#### Nurse-Led Well-Being Interventions

The efficacy of nurse-led interventions was investigated by 4 studies. These interventions generally included a combination of symptom monitoring (or screening) and related tailored support, education about symptom management, and well-being information, which predominantly included signposting rather than specific psychological support. Delivery methods included telephone calls [67-69,71] and web- or app-based programs [69,71]. All programs emphasized symptom management or emotional support via scheduled nurse check-ins, with [71] or without [67-69] additional internet-based information.

QoL outcomes were assessed in 3 studies. All found a positive impact on QoL; although each approach differed, and different outcome measures were used. Specifically, 1 study [68] found that in comparison to controls, patients using nurse-led interventions had higher QoL scores on functional, psychological, and social subscales (with 1 study also finding improved performance in the physical dimensions of QoL [70]). A further noninferiority study [71] found that remote pain-related education and psychological support were as effective as face-to-face therapy for patients with chronic postsurgical pain. While this study failed to report statistics for within-group differences over time, graphs indicate a positive effect on mental health and social functioning following the intervention. However, the quality of this study was rated as poor. Additionally, the good quality RCT by Reinke et al [67] found a consistently positive effect of their intervention on QoL; although this failed to reach statistical significance, this may be because this intervention primarily focused on coordination of care and seemed to lack the psychological coaching or support elements seen in the other studies.

Only 2 studies measured psychological outcomes. One study found a positive effect with reduced anxiety and increased well-being in the IG compared to controls [68]; while a further single-arm pre-post study found that a nurse-led intervention screening for high levels of distress in patients with LC reduced psychological distress through a combination of patient education and tailored coaching calls [69]. However, these studies were rated as being of fair and poor quality, respectively.

#### Multicomponent eHealth Portals and Programs

Finally, 6 studies involved the use of multicomponent eHealth systems that comprised multiple approaches to supporting well-being and QoL, simultaneously [72-76,78]. These interventions were delivered via web-based programs or portals, mobile apps, cloud-based systems, and WeChat programs, and they varied in scope and complexity. While all studies included an element of health-related education, 5 included symptom monitoring [72,74-76,78], 4 included physical activity advice or exercises [73,74,76,78], and 4 included well-being support [72-74,78] (which varied in intensity from preoperative stress reduction to remote counseling). Five studies included at least 3 of these elements within a single program [72-74,76,78].

Five studies evaluated the impact of these tools on QoL [72-76], and all (including 2 good quality RCTs [72,73]) found a positive effect across several QoL domains [72-76]. All were rated as good or fair quality.

Psychological outcomes were measured in 4 studies [72-74,78]. While quality varied from good [72,73] to poor [78], all found a positive impact on psychological well-being. While each study used different measures, anxiety was found to improve in 2 studies [73,74,78]; depression scores improved in 2 [73,74], and a further study found reductions in psychological distress [72].

#### Program Duration

Program duration has previously been identified as a potential predictor of outcome [94]. In the quantitative synthesis, interventions ranged in duration from a single session [47] to up to 1 year [73,75]. Five studies did not specify intervention duration [52,53,60,71,76]. As Figure 2 illustrates, better outcomes were seen for interventions that were 12 weeks or longer in terms of QoL, where 9 of the 12 (75%) studies were found to have a significantly positive impact on patients [48,50,51,54-56,67,68,72-75]. In contrast, only 3 of the 5 (60%) studies with a duration of fewer than 12 weeks were found to have a significantly positive outcome [49,57,59].

In contrast to QoL outcomes, the impact of program duration on psychological outcomes appeared minimal. While 6 of 7 (86%) studies showed consistently positive effects across the different domains when the interventions were 12 weeks or more [54,55,68,72-74], 9 of 11 (82%) studies that were under 12 weeks in duration showed consistently positive effects [49,57,59,61,63,65,66,69,78].

#### Clinician Input

Clinician input was common among the interventions, with most studies in the synthesis (n=29, 97%) facilitating some form of health care professional or patient interaction. These interactions varied in format and included: single face-to-face sessions [47,55], ongoing responses to automated alerts about patient symptomatology [48,49,50-53,75], supervised physical activity [54,60], app or social media-based communication (for example, through WeChat, question and answer forums or other text messaging) [59,63,72-74,78] and telehealth (including both video and audio calls [46,49,50,54,57,59,61,62,64,66-69,71,76]. The 1 study that lacked clinician input found conflicting results, with improvements in QoL noted when using the EQ-VAS, but not the EQ-5D [56].

### Acceptability and Engagement

Across all 33 studies, acceptability of the interventions was quantitatively explored in 42.42% (n=14) of cases. Five studies [49,54,55,77,78] reported that 70%-100% of patients found the interventions to be valuable or helpful, while 4 studies [47,48,54,74] reported that 88%-100% would recommend the intervention. Five studies [58,71,74,76,77] reported 77%-100% of their participants were satisfied with the intervention, while 2 studies [46,61] measured intervention acceptability on self-reported satisfaction scales from 0-4 and found means to be relatively high (all scores were over 3.2). Furthermore, 1 study [66] found that a higher proportion of their participants preferred receiving their intervention online than in person.

Nine studies assessed participants’ engagement with the interventions through metrics such as intervention (or session) completion rates, log-ins, and usage tracking. Engagement levels varied widely. Coats found that patients attended 100% of their activity sessions when they were supervised, and 96% when they were not [58]. In contrast, Bade et al [55] found only 21% of patients met their personalized physical activity goals, although step counts were recorded in 90% of weeks. Granger et al [57] found that only 50% of patients reliably wore their activity trackers, while 62% completed exercise diaries. PRO completion rates varied widely across 3 studies from 40%-82% [48,51,78]. Engagement in psychological well-being interventions seemed generally high, with 2 studies all reporting attendance rates of over 70% [64,66]. For 1 of the multicomponent portal interventions, engagement was mixed. Although 92% of patients logged in more than once and 82% of PROs were completed, the mean number of program log-ins during the 4-month study period was relatively low (<12), as was the mean usage time (<13 minutes) [77].

## Discussion

### Principal Findings

#### Overview

To our knowledge, this is the first systematic review to investigate the potential impact of eHealth interventions on QoL and psychological well-being in patients with LC. The review synthesized findings from 33 studies using eHealth strategies with patients with LC, by first characterizing the different approaches taken and then evaluating their impact.

#### Characterization of Interventions

Several eHealth strategies were identified, which can be categorized into 6 groups based on their modality and theorized mechanism for action. These included:

1. Patient education interventions that are designed to improve QoL by solely focusing on improving health literacy and health-related symptom self-management [95].
2. Symptom monitoring interventions that triggered proactive clinician response when reported symptom values exceeded a certain threshold; likely improving QoL and well-being by enabling earlier identification and timely intervention, preventing deterioration and unplanned hospital admissions, enhancing patient-clinician communication, and empowering patients in self-management of symptoms [96,97].
3. Physical activity and pulmonary rehabilitation interventions that used technology to deliver exercise programs and track progress toward fitness goals (eg, through mobile apps with wearable pedometers). This type of intervention is theorized to impact QoL by improving symptoms (such as dyspnea and cancer-related fatigue), physical function (by enhancing cardiorespiratory fitness and muscle strength), supporting psychological well-being via neuroendocrine and immunological mechanisms (including the release of endorphins and reduction of cortisol), and modulating systemic inflammation [98].
4. Psychological interventions that (while varied in modality and approach) aimed to improve well-being by predominantly targeting cognitive and emotional regulation processes.
5. Nurse-led interventions that used a combination of the abovementioned approaches to support well-being and QoL, often through scheduled or asynchronous support. While the heterogeneous nature of this class of intervention makes it difficult to specify an exact theorized mechanism of action, they were broadly informed by symptom management theory [99].
6. Finally, multicomponent eHealth portals and programs that typically included some combination of interactive features, such as symptom logging, alerts or notifications, written text or multimedia education, guided exercise videos, synchronous or asynchronous communication with health care professionals or researchers, personalized or prespecified physical activity or pulmonary rehabilitation exercise plans, or tailored well-being support.

These interventions likely improve QoL and promote well-being by providing integrated access to multiple supportive approaches simultaneously, providing timely, multipronged, personalized support that helps patients to manage physical and psychological symptoms, increase their health-related knowledge, reduce uncertainty, and enhance self-efficacy [100,101].

#### Impact on QoL

Digital interventions targeting QoL outcomes were examined in 25 studies, which collectively used 7 different validated QoL instruments—most frequently the EORTC QLQ-C30 and the Functional Assessment of Cancer Therapy suite. This diversity in outcome measures complicates direct comparisons across studies but reflects the multidimensional nature of QoL in LC. Despite this heterogeneity, over two-thirds of the studies reported improvements in at least some QoL domains.

Study quality was mixed, with only 9 rated as good, 9 as fair, and 4 as poor. Importantly, 8 of the good-quality studies showed positive effects on QoL, reinforcing confidence in the potential benefit of eHealth interventions in this domain.

The quantitative synthesis of 22 studies found that the most consistent benefits were observed in nurse-led (3/3) and multicomponent (5/5) interventions. These findings broadly confirm those of previous systematic reviews with other cancer populations that have found that the most effective eHealth interventions for improving QoL are multifaceted platforms that integrate diverse resources to address multiple needs across different QoL domains [102]. Symptom monitoring (4/6) and physical activity (4/6) interventions also showed predominantly positive outcomes. These findings are in line with previous reviews that have shown the QoL benefits of pulmonary rehabilitation eHealth interventions with patients with LC [37], and the effectiveness of ePRO-based interventions on symptom burden, QoL, and survival in other cancer populations [102,103].

Conversely, there was little evidence for the impact of patient education programs. This is in contrast with previous work that has found health-related patient education interventions to play an important role in supporting the QoL of patients with chronic health conditions and cancer [104]. However, the 2 studies in this category focused solely on presurgical preparation, making it difficult to conclude the efficacy of educational eHealth strategies in a wider context. Furthermore, patient education was a key element in many multicomponent and nurse-led interventions, suggesting it may be more impactful when integrated into a broader support strategy.

Effective QoL-focused interventions typically lasted at least 12 weeks and included features such as personalized feedback, clinician involvement, and opportunities for sustained patient engagement. In contrast, shorter interventions or those lacking follow-up mechanisms (eg, single-session education) tended to show limited or no improvements in QoL. These findings echo those of a previous meta-analysis [101], highlighting the importance of ongoing patient interaction and tailored support in driving improvements in QoL. However, program duration alone is unlikely to drive positive outcomes. For instance, one 4-month intervention that forms part of this review but was not included in the quantitative synthesis [77] found no significant impact on patient QoL. This is likely due to low engagement, as participants could access the program without active commitment. This suggests that meaningful engagement, rather than duration alone, is critical for intervention effectiveness.

#### Impact on Psychological Well-Being

The reviewed studies also explored the impact of eHealth interventions on psychological aspects of well-being, with more mixed results. Psychological outcomes (spanning anxiety, depression, and distress) were assessed using a wide array of validated tools. In total, 10 different psychological measures were used across 21 studies, contributing to high measurement variability.

Of those included in the quantitative synthesis (n=20), most studies (17/20) found significant improvements in at least one psychological domain, although the strength and consistency of effects varied. As with QoL, nurse-led programs (2/2) and multicomponent digital platforms with embedded emotional support (4/4) yielded the most consistent effects, suggesting multifaceted interventions that can be personalized and tailored to individual needs (either by the deliverer of the intervention, or by the end user) may be particularly beneficial for improving psychological well-being. Furthermore, physical activity (4/4) interventions were also found to be consistent in their positive effects, supporting previous work that has found physical activity to be linked to well-being outcomes in the general population [5,105] and reduced depression symptoms in wider cancer populations [106].

Psychological interventions showed more mixed results, with just over half (3/5) reporting consistently positive outcomes. Outcomes varied according to the different theoretical approaches they took. For example, mindfulness-based interventions demonstrated consistently positive results; a finding that is also reflected across different cancer populations [107]. However, interventions using CBT or ACT delivered via telephone failed to demonstrate consistent improvements. This is at odds with previous work that has found a positive impact of internet-based CBT in other cancer populations [108], and general population studies that have demonstrated the efficacy of computerized CBT and ACT on psychological symptoms [109,110]. However, rather than indicating these psychological strategies may be inappropriate with patients with LC, the null findings may be more reflective of delivery format (telehealth versus online platforms), low baseline symptom levels, or study design limitations including low statistical power and use of active comparators [62,64].

Surprisingly, and in contrast to previous research (eg, [111]), little support was found for the impact of symptom monitoring (1/3) interventions on psychological well-being. The reason for this discrepancy is unclear; however, it may be due to the underpowered nature of these studies. Furthermore, levels of engagement with these programs were rarely measured; thus, a lack of engagement or adherence may also explain these findings.

Again, study quality was mixed: only 8 were rated as good quality, while a further 7 were rated as fair, and 5 as poor. In this case, 5 of the good-quality studies found evidence of a positive impact on psychological well-being. The variation in study quality likely reflects that most were pilot or feasibility studies, underscoring that research into the psychological impact of eHealth in LC care is still in its early stages, and demonstrating the need for more rigorous trials in this area.

While the impact of eHealth on psychological outcomes was varied (a finding not uncommon with patients with cancer [112]), certain intervention parameters were associated with better outcomes than others. For example, interventions that offered structured support over a sustained period, integrated emotional content with practical tools, encouraged continued use and engagement, and were delivered through accessible platforms (eg, apps, WeChat, or web-based programs or portals) were most successful. Studies with positive outcomes also tended to include regular clinician guidance or interpersonal contact, suggesting that human connection (whether synchronous or asynchronous) plays a critical role in psychological benefit.

### Implications of Findings

Unlike traditional models of care, which are temporally and geographically constrained, eHealth interventions offer flexible, open, and interactive avenues of support. Indeed, a particular strength of eHealth is its ability to reach people in rural or underserved areas, enabling timely, remote support by overcoming logistical and health care access-related barriers related to location, staffing shortages, limited access to specialist care, etc [113]. Findings from both this review and the wider literature support the potential benefits of eHealth in cancer care. For instance, several reviews have found eHealth programs to be superior to standard care in terms of improving QoL and psychological outcomes in patients with a range of different cancers [94,100,101,114], with the caveat that the impact on psychological outcomes is less clear.

Notably, intervention modality and design appear to influence outcomes. As also reported by Li et al [94], app- and web-based interventions tend to be more effective than those delivered by telephone. Similarly, our review found that multimodal, platform-based, or nurse-led interventions were consistently more successful than single-focus interventions, aligning with findings from breast cancer populations [102,115]. Given the complex and multifactorial nature of QoL, which can be influenced by disease type, stage, treatment burden, and individual needs [116], these interventions may be particularly successful because they support patients in multiple ways and use personalized tools and solutions to address an array of QoL and well-being needs.

These findings have important real-world care implications, as a recent review found that unmet physical and psychological care needs significantly impact the QoL and well-being of people with LC, highlighting a need for targeted services and interventions to address these needs throughout the disease trajectory [117]. Furthermore, using eHealth strategies to deliver support aligns with the NHS (National Health Service) 10 Year Health Plan [118]—a strategy for England’s NHS aimed at sustainably improving patient care by harnessing the use of digital technology in health care delivery, focusing on illness prevention, the expansion of mental health and well-being support, and emphasizing the importance of community care (ie, shifting care closer to people’s homes rather than in hospitals where possible). However, despite the promise of these tools, real-world implementation remains limited. Most eHealth interventions are not yet integrated into routine care, and when they are, they are often not used as intended, reducing their potential benefit [119,120].

Barriers to implementation at the institutional level and challenges to uptake among both health care professionals and patients require further investigation and targeted solutions. One key consideration is the feasibility of delivering eHealth interventions, particularly those that involve ongoing clinician involvement. Several interventions reviewed in the current paper included scheduled, individualized contact over extended periods (eg, [73]), which raises concerns about cost and scalability and may hinder wider implementation. Thus, cost analyses need to be carried out to assess the feasibility of their delivery. From the user perspective, although acceptability within this review was generally reported as high and many participants found these interventions helpful, this was assessed in only a minority of studies and warrants deeper exploration. While prior research has shown high satisfaction with digital health tools in chronic illness populations [121], actual engagement with such programs can be inconsistent. This may be influenced by factors such as lack of trust in digital systems, poor digital literacy, and minimal tailoring to age, gender, or disease stage [112,114]. To address these issues, future research should prioritize strategies to enhance engagement, such as patient and public involvement. Only 4 studies in the current review discussed how their interventions were developed (ranging from top-down development by experts to patient and public involvement co-design), so there is limited evidence as to how these strategies may have impacted acceptability and engagement. However, previous work suggests that taking an expert-by-experience approach can hold significant potential to improve both uptake and sustained use of interventions [122].

### Key Strengths and Limitations of This Study

There are several strengths associated with this review, including a rigorous and systematic search and selection process that ensured comprehensive evidence coverage of the literature and the application of a validated quality assessment tool. Additionally, previous work has emphasized the need for research to investigate the components and features of eHealth programs that lead to improved therapeutic outcomes [115]. A further strength of this review is that it aimed to achieve this by characterizing the nature of the eHealth interventions and strategies used with patients with LC and elucidating the approaches that are likely to positively impact QoL and psychological well-being. However, we acknowledge that when combination approaches are taken, it is difficult to ascertain the specific aspects of the interventions that are driving the outcomes, particularly as the included multicomponent studies did not specify which aspects of their interventions resulted in change.

Several limitations should also be acknowledged when interpreting our results. First, our search was restricted to studies published in English and did not include gray literature, potentially omitting relevant research. Second, we found significant heterogeneity across the included studies in our review, due to variations in study designs, outcome measures used, intervention types, intervention lengths, follow-up periods, and control conditions (when included). The absence of homologous methods and approaches across studies prevented a meaningful meta-analysis. As such, this review relied on a predominantly narrative synthesis based on vote counts of effect directions. While this approach can indicate whether an effect exists, it cannot estimate the combined significance of the studies or the magnitude of the effects. These limitations may reduce the strength of the conclusions that can be drawn, but also highlight the need for more standardized reporting in future primary studies.

Third, many digital tools used in health care settings (such as patient portals, electronic records, and well-being tools) are developed for broader populations and not tailored specifically to those with cancer. As the current review focused only on interventions designed for people living with or beyond cancer, we may have missed a wider range of potentially relevant tools. As such, the total number and diversity of eHealth options for patients with LC may be larger than currently represented in the review.

Finally, review methods aside, the limitations of the included literature must also be noted. The quality of studies included in the review was variable, with no studies rated as being of excellent quality. Additionally, many of the studies were pilot, feasibility, or single-arm studies with associated risks of bias, and many had small sample sizes (of *n*<50), limiting their generalizability. Additional large-scale RCTs using standardized QoL and well-being measures are needed to strengthen the evidence for the efficacy of these interventions. Furthermore, due to the relatively short follow-up periods in this review, evidence for the long-term effects of eHealth interventions was limited.

### Conclusions

This review presents the first comprehensive synthesis of evidence on the role of eHealth in supporting QoL and psychological well-being among people living with LC. The findings suggest that multicomponent and nurse-led digital interventions, particularly those that are personalized, involve clinician input, and are delivered via apps or web-based platforms, consistently demonstrate positive effects across both QoL and psychological domains. Additionally, longer-duration interventions (12 weeks or more) were particularly helpful for QoL. Physical activity programs were also frequently effective for both QoL and psychological well-being. However, while symptom monitoring programs had a positive impact on QoL, their effects on well-being were less clear, potentially due to small sample sizes or implementation issues. Overall, eHealth interventions show strong promise as acceptable, scalable, and flexible tools for addressing the complex and varied supportive care needs of people living with LC; yet, their integration into routine care remains limited, and few are tailored specifically to this population.

While the findings of this review are encouraging, variability in study quality, design, and outcome measures, along with limited real-world implementation, underscores the need for more rigorous, standardized research. To fully realize the potential of eHealth in this context, future efforts must focus on the development of well-designed, targeted digital solutions, with clear strategies for implementation, sustained patient engagement, and meaningful involvement of people with lived experience. Doing so could help close existing gaps in care, improve QoL and well-being, and contribute to the broader goals of delivering accessible, personalized cancer care.

## Appendix

Multimedia Appendix 1 PRISMA checklist. PRISMA: Preferred Reporting Items for Systematic Reviews and Meta-Analyses.

## Abbreviations

ACT: acceptance and commitment therapy
CBT: cognitive behavioral therapy
EORTC QLQ-C30: European Organisation for Research and Treatment of Cancer Quality of Life Questionnaire
ESAS: Edmonton Symptom Assessment System
IG: intervention group
LC: lung cancer
NHS: National Health Service
PRISMA: Preferred Reporting Items for Systematic Reviews and Meta-Analyses
PRO: patient-reported outcome
PROSPERO: International Prospective Register of Systematic Reviews
QoL: quality of life
RCT: randomized controlled trial
